# Supplementary material for: Identifying context-specific domains for assessing antimicrobial stewardship programmes in Asia: protocol for a scoping review
Source: BMJ Open. 2022 Sep 14;12(9):e061286. doi: 10.1136/bmjopen-2022-061286 (PMC9478836; doi:10.1136/bmjopen-2022-061286)
Supplement: Author's manuscript [file bmjopen-2022-061286.draft_revisions.pdf]

BMJ Open is committed to open peer review. As part of this commitment we make the peer review history of every article we publish publicly available.

When an article is published we post the peer reviewers' comments and the authors' responses online. We also post the versions of the paper that were used during peer review. These are the versions that the peer review comments apply to.

The versions of the paper that follow are the versions that were submitted during the peer review process. They are not the versions of record or the final published versions. They should not be cited or distributed as the published version of this manuscript.

BMJ Open is an open access journal and the full, final, typeset and author-corrected version of record of the manuscript is available on our site with no access controls, subscription charges or pay-per-view fees (http://bmjopen.bmj.com).

If you have any questions on BMJ Open's open peer review process please email info.bmjopen@bmj.com

BMJ Open

Identifying context-specific domains for assessing antimicrobial stewardship programs in Asia: Protocol for a scoping review

Journal:	*BMJ Open*
Manuscript ID	bmjopen-2022-061286
Article Type:	Protocol
Date Submitted by the Author:	20-Jan-2022

Complete List of Authors:	Vu, Huong; Oxford University Clinical Research Unit<br>Hamers, Raph ; University of Oxford, Eijkman-Oxford Clinical Research Unit; University of Oxford, Centre for Tropical Medicine and Global Health, Nuffield Department of Medicine<br>Limato, Ralalicia; University of Oxford, Eijkman-Oxford Clinical Research Unit; University of Oxford, Centre for Tropical Medicine and Global Health, Nuffield Department of Medicine<br>Limmathurotsakul, Direk; Mahidol Oxford Tropical Medicine Research Unit; University of Oxford, Centre for Tropical Medicine and Global Health, Nuffield Department of Medicine<br>Karkey, Abhilasha; University of Oxford Nuffield Department of Clinical Medicine, Centre for Tropical Medicine and Global Health; Oxford University Clinical Research Unit, Nepal Unit<br>Dodds Ashley, Elizabeth; Duke University, Duke Antimicrobial Stewardship Outreach Network, Duke Center for Antimicrobial Stewardship and Infection Prevention<br>Anderson, D; Duke University, Duke Antimicrobial Stewardship Outreach Network, Duke Center for Antimicrobial Stewardship and Infection Prevention<br>Patel, Payal ; Centers for Disease Control and Prevention<br>Patel, Twisha ; Centers for Disease Control and Prevention<br>Lessa, Fernanda; Centers for Disease Control and Prevention<br>van Doorn, H. Rogier; Oxford University Clinical Research Unit; University of Oxford Nuffield Department of Clinical Medicine

Keywords:	HEALTH SERVICES ADMINISTRATION & MANAGEMENT, INFECTIOUS DISEASES, PUBLIC HEALTH

SCHOLARONE™
Manuscripts

Manuscript Title: Identifying context-specific domains for assessing antimicrobial stewardship programs in Asia: Protocol for a scoping review

Authors: Vu Thi Lan Huong[1]*, Raph L. Hamers[2,3], Ralalicia Limato[2,3], Direk Limmathurotsakul[3,4], Abhilasha Karkey[3,5], Elizabeth Dodds-Ashley[6], Deverick Anderson[6], Payal K. Patel[7], Twisha S. Patel[7], Fernanda C. Lessa[7], H Rogier van Doorn[1,3]

Affiliations:

[1]Oxford University Clinical Research Unit – Ha Noi, Viet Nam, 78 Giai Phong – Dong Da – Ha Noi – Viet Nam

[2]Eijkman-Oxford Clinical Research Unit – Jakarta, Indonesia

[3]Centre for Tropical Medicine and Global Health, Nuffield Department of Medicine, University of Oxford, Oxford, UK

[4]Mahidol Oxford Tropical Medicine Research Unit – Bangkok, Thailand

[5]Oxford University Clinical Research Unit - Kathmandu, Nepal

[6]Duke Antimicrobial Stewardship Outreach Network, Duke Center for Antimicrobial Stewardship and Infection Prevention, Duke University, Durham, NC 27710, United States

[7]Division of Healthcare Quality Promotion, US Centers for Disease Control and Prevention, USA

Correspondence to Dr Vu Thi Lan Huong: huongvtl@oucru.org

CDC disclaimer: The findings and conclusions in this report are those of the author(s) and do not necessarily represent the official position of the U.S. Centers for Disease Control and Prevention

For peer review only

Abstract

Introduction

Antimicrobial stewardship (AMS) is an important strategy to combat antimicrobial resistance. Resources are available to provide guidance for design and implementation of AMS programs, however these may have limited applicability in resource-limited settings including those in Asia. This scoping review aims to identify context-specific domains and items for the development of a healthcare-facility (HCF)-level tool to guide AMS implementation in Asia.

Methods and analysis

This review is the first step in a larger project to assess AMS implementation, needs and gaps in resource-limited regions in Asia. We will employ a deductive qualitative approach to identify locally appropriate domains and items of AMS implementation guided by Nilsen & Bernhardsson's contextual dimensions. This process is also informed by discussions from a technical advisory group coordinated by the U.S. Centers for Disease Control and Prevention to develop an AMS HCF-level assessment tool for low- and middle-income countries (LMIC). We will review English-language documents that discuss HCF-level implementation, including those describing frameworks, components/elements, or recommendations for design, implementation or assessment globally and specific to Asia. We will search general electronic databases (MEDLINE, Embase, Web of Science and Google Scholar), region-specific databases, AMS national action plans, grey literature sources and reference lists to identify eligible documents. Country-specific documents will be restricted to countries in three sub-regions: South Asia, East Asia and Southeast Asia. Codes and themes will be derived through a content analysis, classified following the predefined context dimensions and used for developing domains and items of the assessment tool.

Ethics and dissemination

Results from this review will feed into our stepwise process for developing a context-specific HCF-level assessment tool for AMS programs to assess the implementation status, identify intervention opportunities and monitor progress over time. The process will be done in consultation with local stakeholders, the end-users of the generated knowledge.

For peer review only - http://bmjopen.bmj.com/site/about/guidelines.xhtml

Strengths and limitations of this review

- This scoping review will be guided by determinant frameworks that identify the context dimensions of implementation and by experts and current research to provide comprehensive and in-depth description of the relevant aspects of AMS programs in general and in Asia;
- We will employ a combination of search strategies that includes different types of databases to efficiently identify a broad spectrum of guidance documents from published and grey literature in English and four local Asian languages;
- Local stakeholders who are the end-users of this generated knowledge will be engaged during the review process;
- We will not include original studies that reported results of AMS implementation from a limited number of single institutions or locations (due to the vast number of such reports)

For peer review only

For peer review only - http://bmjopen.bmj.com/site/about/guidelines.xhtml

Introduction

The prevalence and incidence rates of antimicrobial resistant (AMR) infections continue to rise, with higher levels of resistance reported from low-and middle-income countries (LMIC) compared to high-income countries. [1] Misuse and overuse of antimicrobials in healthcare continues to be one major driver as consumption is accelerating worldwide especially in emerging economies. At the same time, access to life-saving drugs targeting the emerging resistant pathogens remains an issue in these settings while development of new drugs has been slow especially for the antibiotics against gram-negative pathogens. [2]

In this context, antimicrobial stewardship (AMS) is an important strategy in global and national action plans to combat AMR. This has been defined in a number of ways from technical descriptions focusing on prescriptions (e.g., drug, dose, duration) to the concepts of responsibility in how antibiotics are used (e.g., careful and responsible decisions to treat). [3] AMS has also been expanded beyond its core strategies and adult inpatient settings, with increasing roles of front-line providers (e.g., nurses and pharmacists), rapidly evolving metrics for impact and success, and wide spectrum of implementation, engagement and practice. [4]

Guidance for AMS programs are available from many resources such as those from World Health Organization (WHO) and the U.S. Centers for Disease Control and Prevention (CDC) [5-7], expert reviews [8 9], country and region specific guidelines. The core elements and components of AMS programs identified by several groups [10-13] often share similar aspects including senior management commitment including resource allocation, leadership including accountability, expertise on infection management and antimicrobials, specific actions to improve antimicrobial use, education and training, monitoring use and resistance epidemiology and reporting and feedback. These existing guidance and tools are usually not context specific, not tailored for resource-limited settings, and not focused on implementation aspects of the programs. The WHO toolkit for LMICs issued in 2019 and WHO policy guidance on integrated antimicrobial stewardship activities issued in May 2021 discuss different sets of measures/indicators for assessing program impact at the healthcare facility (HCF) level [7 14], which include structural, process and outcome measures but these assessments are at a high level and do not include specific implementation contextual details of stewardship interventions in these settings. Therefore, existing

For peer review only - http://bmjopen.bmj.com/site/about/guidelines.xhtml

guidance resources should be reviewed to develop a healthcare-facility (HCF)-level tool that will guide implementation of AMS in hospitals in Asia.

Review rationale

The existing guidance documents may have limited applicability to many countries in Asia where healthcare systems, the availability of diagnostic testing and antibiotics, public awareness and prescribing practices [8 15], and contextual and cultural characteristics [16 17] differ from the other regions. Therefore, contextualization of the assessment of the status of AMS program implementation is important. A "glocalization" approach has been discussed for reconciliation of global frameworks and methodologies and the local contexts in AMS implementation [18]. In November 2016, an expert panel consisting of 11 infectious diseases experts, researchers and opinion leaders from Asia developed a consensus statement on AMS programs for acute care hospitals in the Asian region [19]. The panel identified common gaps and challenges facing AMS programs, potential solutions, and process and outcome measures for evaluating impact of the programs. A checklist was designed based on existing materials. However, it provides limited in-depth understanding of the current state to support effective planning of targeted interventions. In May 2021, WHO issued a policy guidance on integrated AMS activities [14]. Within this guidance, WHO developed a list of essential national and HCF core elements to assist with strengthening AMS structures at both levels. Context-specific matters for HCF-level AMS implementation are not yet captured in the HCF tool. Understanding implementation needs and practices within the Asian context and being able to monitor progress over time are key to assist local healthcare facilities with improving antimicrobial use. A context-specific assessment tool that is practical and responsive to the local conditions is needed to support institutions to start and maintain AMS programs in Asia. This assessment tool will be complementary to the WHO tools on national and HCF-level to provide support for hospitals in assessing their AMS status and improving the program implementation.

Review objectives

This scoping review protocol aims to describe our methods to identify the context-specific domains and items to develop a tailored assessment tool for Asia. Existing guidance documents will be analyzed to extract these contextual domains and items related to an AMS program in general and specific to Asian settings. This work constitutes the first step in a stepwise approach to the development of an integrated

For peer review only - http://bmjopen.bmj.com/site/about/guidelines.xhtml

assessment tool that can be used to assess the current practices and needs and identify priorities for implementation and improvement of AMS programs in Asia.

Methods and analysis

Conceptual model

To guide our data extraction and understanding of contextual features in AMS implementation, we will use the four levels of contextual dimensions (micro, meso, macro, and multiple) as identified by Nilsen & Bernhardsson in their review of contextual determinants for implementation outcomes from 17 determinant frameworks [20] listed Table 1. We will employ a deductive qualitative approach to identify the domains and items of AMS implementation according to the contextual dimensions. This process is also informed by the discussions from a technical advisory group coordinated by the U.S. CDC that has been formed to develop a HCF assessment tool focused on AMS implementation.

Table 1. List of contextual dimensions for program implementation to guide the scoping review of AMS guidance documents

Dimension	Description of initial themes and codes for data extraction
Micro level of healthcare	Patients' factors (knowledge, attitudes, preferences, needs, and resources in antibiotic use and AMR)
Meso level of healthcare	Organizational culture and climate, readiness to change (commitment, preparation, prioritization, efficacy and capacity to change, tension, practicality, flexibility), support (administration, staff, training resources, information and decision-support systems, expert support), structures (size, complexity, specialization, differentiation, decentralization)
Macro level of healthcare	Exogenous influences (policies, guidelines, research evidence, regulation and legislation, mandates, directives, recommendations, political stability, public reporting, benchmarking, organizational networks)
Multiple levels of healthcare	Social relations and support, financial resources (funding, reimbursement, incentives, costs), leadership (leaders, champions…), time restrictions, feedback (mechanisms for

	monitoring and providing feedback), physical conditions (equipment, facilities)

Protocol design

This protocol follows the methodology for scoping review developed by Arksey and O'Malley [21] and Levac *et al* [22] through six steps:

Stage 1: Identifying the research question

This scoping review is designed to answer the following question: "What are the domains and items that can constitute an assessment tool of AMS implementation in Asia at a HCF level?" The assessment tool will aim to assess the state of AMS implementation in healthcare facilities and contextual factors can influence its success and impact. It will also aim to identify challenges and opportunities, and help formulate priorities by generating actionable recommendations for next steps in the implementation of AMS programs.

Stage 2: Identifying relevant documents

We will search a number of electronic databases of published and unpublished literature to identify documents that provide guidance on AMS implementation globally and specific to Asia (Table 2). Country-specific documents will be restricted to the countries within the three sub-regions of Asia: South Asia, East Asia and Southeast Asia. After this search, the study investigator group will review the search results to determine if documents identified represent the available AMS guidance resources commonly used. The search will be expanded to additional search engines available and manually checking all reference lists of included documents will also be performed to identify additional documents of relevance. We will also conduct a targeted search of the grey literature in local and international organisations' websites.

Table 2. List of databases to be searched for the scoping review

Database category	Databases
General database (a recommended optimal combination for searching systematic reviews [23])	Embase<br>MEDLINE (including "Epub Ahead of Print, In-Process, and Other Non-Indexed Citations")<br>Web of Science (Core Collection)<br>Google Scholar (first 200 references of relevance)

For peer review only

Regional database	China: Chinese biomedical literature Database (CBM)<br>India: IndMED<br>Korea: KoreaMed<br>South-east Asia: Index Medicus for the South-East Asia Region (IMSEAR)<br>China National Knowledge Infrastructure (CNKI)<br>Chinese Scientific Journal Database (VIP)<br>SaudiMedLit<br>Thai Index Medicus<br>Thai Journal Citation Index Centre<br>Thai Medical Index<br>Western Pacific Region Index Medicus (WPRIM)<br>WANFANG Data<br>Korea: RISS- Korean Education and Research Information Service<br>Asia: Bibliography of Asian Studies<br>Health Research and Development Information Network (HERDIN)<br>Japan: Cinii<br>Indonesia: Garba Rujukan Digital, Neliti
Grey literature database	ProQuest Dissertation and Theses Database (PQDT)<br>OpenGrey<br>Grey literature report

Following search terms will be used for the general databases (Embase MEDLINE, Web of Science, Google Scholar) as both keywords in the title and/or abstract and subject headings (eg, MeSH, EMTREE) as appropriate: (“antimicrobial stewardship” OR “antibiotic stewardship”) AND (“framework” OR “guid*” OR “tool*” OR “recommend*” OR “step” OR “approach” OR “policy”). For other databases, we will search using less restrictive terms (“antimicrobial stewardship” OR “antibiotic stewardship”) to be able to identify local relevant documents. Only documents published in English language will be included, with no restriction on years of publication.

Stage 3: Selection of documents for review

For peer review only - http://bmjopen.bmj.com/site/about/guidelines.xhtml

This stage will be an iterative process: searching, refining the search strategy, and reviewing articles for eligibility and inclusion into the final list of documents. The team will meet to discuss inclusion and exclusion criteria at the beginning of the process, and to review challenges and uncertainties and to refine the search strategy as needed along the process. The review process will essentially involve two steps. In the first step, two research assistants will independently screen the titles and abstracts of all citations returned from the search databases. Any documents considered relevant by either or both researchers will be included in the list for full-text screening at this step. In step two, the lead investigator (VTLH) together with the two research assistants will review the full-text of these documents and select those that fully meet inclusion/exclusion criteria for data extraction stage. Any discrepancies the documents selected arise during the process will be discussed, and decision will be made through consensus within the team.

Documents will be included if they provide guidance for and/or input information that can help inform AMS implementation at the HCF level, including those describing frameworks, program components and elements, or recommendations for design, implementation or assessment. We will exclude any documents that only describe methods or results of research studies or reports on AMS programs implemented at single healthcare facilities with a limited geographical scope. We will also exclude documents without identifiable authors, publishers, or year of publication. For guidance documents from the same institutions that were updated over time, we will only include the most updated version in the review.

Stage 4: Data extraction

Characteristics of included documents will be extracted using a data extraction form, which will be reviewed and updated along the extraction process as appropriate. Following information will be captured: confirmation on document's inclusion/ exclusion criteria, publication year, geographical scope (global or regional), country, type of document (guidance, review, perspective/commentary, others), author, publisher and type (government agency, international body, expert group, individual authors, not-for-profit organization, for-profit organization, others). Data extraction will be based on the content published, no attempts will be made to contact authors for clarifications.

For peer review only

The content of the included documents will be read in-depth and coded following the context dimensions described by Nilsen & Bernhardsson. Initial themes and codes were developed based on the contextual dimensions as described in Table 1. Codes are the specific summarizing phrases; each code presents one specific area of AMS implementation that should be assessed. Themes are the higher-order categories consisting of multiple codes, each theme represents one broad area or construct of AMS implementation. The initial structure of themes and codes can be expanded during the extraction to add additional codes and themes and can be re-structured if needed for clarity. We will use a deductive qualitative content analysis [24] approach that allows the analysis to evolve from our existing understanding of AMS implementation and the existing research about program implementation. We will use NVivo software, a package for text-based analysis, to manage the texts and systematically organize our reading and coding. The qualitative content analysis is a popular, flexible, and pragmatic method of analyzing text data to attain a condensed and broad description of the topic through a systematic classification process involving coding and identifying themes and patterns [24]. The coding process will be conducted with close supervision by two experienced researchers of the research team. The research team will meet regularly to review the codes identified and ensure data extraction is consistent with the research question and purpose.

Stage 5: Data summary and synthesis of results

After the documents were read and coded, we will summarize and organize the codes and themes into a cohesive and coherent structure. From this, we will identify possible items and domains that will be used to develop a HCF-level AMS assessment tool of AMS implementation. One item can be identified from the content of multiple codes and presented in a question format. One domain can be identified from the content of multiple themes that can help describe the items under it. Through this scoping review process, we will be able to achieve a broad and in-depth description of the various aspects of AMS implementation that are applicable to Asian settings. This will help identify aspects commonly encountered in an AMS program, but also point out the region-specific issues, challenges and opportunities and help the development of a practical and responsive assessment tool for use in these settings.

Stage 6: Consultation

For peer review only - http://bmjopen.bmj.com/site/about/guidelines.xhtml

To provide insights into the process, we will engage local stakeholders including the hospitals personnel involved with AMS who will be the end-users of the knowledge generated from this scoping review. Local stakeholders will be identified through our own individual networks used to identify the local documents that are not available in the published literature. The feedback from these local experts on the list of domains and items will be captured using a web-based survey.

Patient and Public Involvement

Patients or the public will not be involved in this scoping review. The assessment domains and items identified from this scoping review will be sent to local experts in antimicrobial stewardship for their feedback on the relevance of these to be included in the assessment tool at healthcare facility level.

Dissemination and ethics

This scoping review is the first step in the overall project coordinated by the U.S. CDC to assess AMS implementation in LMICs including Asia. The results from this scoping review will be used to develop a context-specific, assessment tool aiming at characterizing the current state of AMS implementation and identifying interventions and opportunities for improvement. The scoping review process will only involve identification, selection and analysis of documents available for use in the public domains, therefore an ethics approval is not required in this study. The whole process of assessment tool development will be done in consultation with local stakeholders who are the end-users of the generated knowledge.

Contributors: VTLH conceived of the idea, developed the research question and study methods. RLH, RL, DL, AK, EDA, DA, PKP, TSP, FCL and HRvD reviewed and aided in developing the research question and study methods. HRvD supervised the work throughout. All authors contributed meaningfully to the drafting and editing, and approved the final manuscript.

Funding: This work is part of the project entitled “Understanding variations in antimicrobial stewardship (AMS) programs in hospital networks in Asia through a newly developed context-specific tool” funded by U.S. Centers for Disease Control

For peer review only - http://bmjopen.bmj.com/site/about/guidelines.xhtml

and Prevention (CDC) through the Broad Agency Announcement (BAA) 75D301-21-R-71738. HVTL was also supported by the National Institute for Health Research (NIHR) (using the UK's Official Development Assistance (ODA) Funding) and Wellcome (Grant Reference Number: 216367/Z/19/Z) under the NIHR-Wellcome Partnership for Global Health Research. The views expressed are those of the authors and not necessarily those of Wellcome, the NIHR or the Department of Health and Social Care.

Competing interests: None declared.

Data sharing statement: No additional data are available.

ORCID IDs:

Vu Thi Lan Huong 0000-0002-9579-5576

H. Rogier van Doorn 0000-0002-9807-1821

Raph L. Hamers 0000-0002-5007-7896

Direk Limmathurotsakul 0000-0001-7240-5320

Ralalicia Limato 0000-0002-5306-3254

For peer review only

References

1. Temkin E, Fallach N, Almagor J, et al. Estimating the number of infections caused by antibiotic-resistant *Escherichia coli* and *Klebsiella pneumoniae* in 2014: a modelling study. *Lancet Glob Health* 2018;6(9):e969-e79.

2. Sriram A, Kalanxhi E, Kapoor G, et al. State of the world's antibiotics 2021: A global analysis of antimicrobial resistance and its drivers. Washington DC.: Center for Disease Dynamics, Economics & Policy, 2021.

3. Dyar OJ, Huttner B, Schouten J, et al. What is antimicrobial stewardship? *Clin Microbiol Infect* 2017;23(11):793-98.

4. Emberger J, Tassone D, Stevens MP, et al. The Current State of Antimicrobial Stewardship: Challenges, Successes, and Future Directions. *Current infectious disease reports* 2018;20(9):31.

5. Centers for Disease Control and Prevention (CDC). Core Elements of Hospital Antibiotic Stewardship Programs, https://www.cdc.gov/antibiotic-use/core-elements/hospital.html. 2019

6. Centers for Disease Control and Prevention. Core elements of antibiotic stewardship programs in resource-limited settings. Atlanta, GA, 2018.

7. World Health Organization. Antimicrobial stewardship programmes in health-care facilities in low- and middle-income countries: a WHO practical toolkit. Geneva: World Health Organization, 2019.

8. Resman F. Antimicrobial stewardship programs; a two-part narrative review of step-wise design and issues of controversy Part I: step-wise design of an antimicrobial stewardship program. *Ther Adv Infect Dis* 2020;7:2049936120945083.

9. Mendelson M, Morris AM, Thursky K, et al. How to start an antimicrobial stewardship programme in a hospital. *Clin Microbiol Infect* 2020;26(4):447-53.

10. Pulcini C, Binda F, Lamkang AS, et al. Developing core elements and checklist items for global hospital antimicrobial stewardship programmes: a consensus approach. *Clin Microbiol Infect* 2019;20(1):20-25. doi: 10.1016/j.cmi.2018.03.033

11. Pollack LA, Srinivasan A. Core elements of hospital antibiotic stewardship programs from the Centers for Disease Control and Prevention. *Clin Infect Dis* 2014;59 Suppl 3(Suppl 3):S97-100.

12. Pollack LA, Plachouras D, Gruhler H, et al. Summary the modified Delphi process for common structure and process indicators for hospital antimicrobial stewardship programs (Transatlantic Taskforce on Antimicrobiol Resistance). 2015. https://www.cdc.gov/drugresistance/pdf/summary_of_tatfar_recommendation_1.pdf (accessed 16 January 2022).

For peer review only - http://bmjopen.bmj.com/site/about/guidelines.xhtml

13. European Commission. EU Guidelines for the prudent use of antimicrobials in human health. *Official Journal of the European Union* 2017;2017/C 212/01

14. World Health Organization. WHO policy guidance on integrated antimicrobial stewardship activities. Geneva: World Health Organization, 2021.

15. Cox JA, Vlieghe E, Mendelson M, et al. Antibiotic stewardship in low- and middle-income countries: the same but different? *Clin Microbiol Infect* 2017;23(11):812-18.

16. Charani E, Smith I, Skodvin B, et al. Investigating the cultural and contextual determinants of antimicrobial stewardship programmes across low-, middle- and high-income countries-A qualitative study. *PLoS One* 2019;14(1):e0209847.

17. Gebretekle G, Haile Mariam D, Abebe W, et al. Opportunities and barriers to implementing antibiotic stewardship in low and middle-income countries: Lessons from a mixed-methods study in a tertiary care hospital in Ethiopia. *PLoS One* 2018;13(12):e0208447.

18. Rubin O. The glocalization of antimicrobial stewardship. *Global Health* 2019;15(1):54-54.

19. Apisarnthanarak A, Kwa AL-H, Chiu C-H, et al. Antimicrobial stewardship for acute-care hospitals: An Asian perspective. *Infect Control Hosp Epidemiol* 2018;39(10):1237-45.

20. Nilsen P, Bernhardsson S. Context matters in implementation science: a scoping review of determinant frameworks that describe contextual determinants for implementation outcomes. *BMC Health Services Research* 2019;19(1):189.

21. Arksey H, O'Malley L. Scoping studies: towards a methodological framework. *International Journal of Social Research Methodology* 2005;8(1):19-32.

22. Levac D, Colquhoun H, O'Brien KK. Scoping studies: advancing the methodology. *Implementation Science* 2010;5(1):69.

23. Bramer WM, Rethlefsen ML, Kleijnen J, et al. Optimal database combinations for literature searches in systematic reviews: a prospective exploratory study. *Systematic Reviews* 2017;6(1):245.

24. Hsieh H-F, Shannon SE. Three Approaches to Qualitative Content Analysis. *Qualitative Health Research* 2005;15(9):1277-88.

For peer review only

Preferred Reporting Items for Systematic reviews and Meta-Analyses extension for Scoping Reviews (PRISMA-ScR) Checklist

SECTION	ITEM	PRISMA-ScR CHECKLIST ITEM	REPORTED ON PAGE #
**TITLE**			
Title	1	Identify the report as a scoping review.	1
**ABSTRACT**			
Structured summary	2	Provide a structured summary that includes (as applicable): background, objectives, eligibility criteria, sources of evidence, charting methods, results, and conclusions that relate to the review questions and objectives.	2
**INTRODUCTION**			
Rationale	3	Describe the rationale for the review in the context of what is already known. Explain why the review questions/objectives lend themselves to a scoping review approach.	5
Objectives	4	Provide an explicit statement of the questions and objectives being addressed with reference to their key elements (e.g., population or participants, concepts, and context) or other relevant key elements used to conceptualize the review questions and/or objectives.	5-7
**METHODS**			
Protocol and registration	5	Indicate whether a review protocol exists; state if and where it can be accessed (e.g., a Web address); and if available, provide registration information, including the registration number.	NA
Eligibility criteria	6	Specify characteristics of the sources of evidence used as eligibility criteria (e.g., years considered, language, and publication status), and provide a rationale.	7-9
Information sources*	7	Describe all information sources in the search (e.g., databases with dates of coverage and contact with authors to identify additional sources), as well as the date the most recent search was executed.	7-9
Search	8	Present the full electronic search strategy for at least 1 database, including any limits used, such that it could be repeated.	7-8
Selection of sources of evidence†	9	State the process for selecting sources of evidence (i.e., screening and eligibility) included in the scoping review.	9
Data charting process‡	10	Describe the methods of charting data from the included sources of evidence (e.g., calibrated forms or forms that have been tested by the team before their use, and whether data charting was done independently or in duplicate) and any processes for obtaining and confirming data from investigators.	9-10
Data items	11	List and define all variables for which data were sought and any assumptions and simplifications made.	9
Critical appraisal of individual sources of evidence§	12	If done, provide a rationale for conducting a critical appraisal of included sources of evidence; describe the methods used and how this information was used in any data synthesis (if appropriate).	NA

For peer review only - http://bmjopen.bmj.com/site/about/guidelines.xhtml

SECTION	ITEM	PRISMA-ScR CHECKLIST ITEM	REPORTED ON PAGE #
Synthesis of results	13	Describe the methods of handling and summarizing the data that were charted.	10
**RESULTS**			
Selection of sources of evidence	14	Give numbers of sources of evidence screened, assessed for eligibility, and included in the review, with reasons for exclusions at each stage, ideally using a flow diagram.	NA
Characteristics of sources of evidence	15	For each source of evidence, present characteristics for which data were charted and provide the citations.	NA
Critical appraisal within sources of evidence	16	If done, present data on critical appraisal of included sources of evidence (see item 12).	NA
Results of individual sources of evidence	17	For each included source of evidence, present the relevant data that were charted that relate to the review questions and objectives.	NA
Synthesis of results	18	Summarize and/or present the charting results as they relate to the review questions and objectives.	NA
**DISCUSSION**			
Summary of evidence	19	Summarize the main results (including an overview of concepts, themes, and types of evidence available), link to the review questions and objectives, and consider the relevance to key groups.	NA
Limitations	20	Discuss the limitations of the scoping review process.	NA
Conclusions	21	Provide a general interpretation of the results with respect to the review questions and objectives, as well as potential implications and/or next steps.	NA
**FUNDING**			
Funding	22	Describe sources of funding for the included sources of evidence, as well as sources of funding for the scoping review. Describe the role of the funders of the scoping review.	11

JBI = Joanna Briggs Institute; PRISMA-ScR = Preferred Reporting Items for Systematic reviews and Meta-Analyses extension for Scoping Reviews.
* Where *sources of evidence* (see second footnote) are compiled from, such as bibliographic databases, social media platforms, and Web sites.
† A more inclusive/heterogeneous term used to account for the different types of evidence or data sources (e.g., quantitative and/or qualitative research, expert opinion, and policy documents) that may be eligible in a scoping review as opposed to only studies. This is not to be confused with *information sources* (see first footnote).
‡ The frameworks by Arksey and O'Malley (6) and Levac and colleagues (7) and the JBI guidance (4, 5) refer to the process of data extraction in a scoping review as data charting.
§ The process of systematically examining research evidence to assess its validity, results, and relevance before using it to inform a decision. This term is used for items 12 and 19 instead of "risk of bias" (which is more applicable to systematic reviews of interventions) to include and acknowledge the various sources of evidence that may be used in a scoping review (e.g., quantitative and/or qualitative research, expert opinion, and policy document).

From: Tricco AC, Lillie E, Zarin W, O'Brien KK, Colquhoun H, Levac D, et al. PRISMA Extension for Scoping Reviews (PRISMAScR): Checklist and Explanation. Ann Intern Med. 2018;169:467–473. doi: 10.7326/M18-0850.

For peer review only - http://bmjopen.bmj.com/site/about/guidelines.xhtml

BMJ Open

Identifying context-specific domains for assessing antimicrobial stewardship programs in Asia: Protocol for a scoping review

Journal:	*BMJ Open*
Manuscript ID	bmjopen-2022-061286.R1
Article Type:	Protocol
Date Submitted by the Author:	30-Jun-2022
Complete List of Authors:	Vu, Huong; Oxford University Clinical Research Unit<br>Hamers, Raph ; University of Oxford, Eijkman-Oxford Clinical Research Unit; University of Oxford, Centre for Tropical Medicine and Global Health, Nuffield Department of Medicine<br>Limato, Ralalicia; University of Oxford, Eijkman-Oxford Clinical Research Unit; University of Oxford, Centre for Tropical Medicine and Global Health, Nuffield Department of Medicine<br>Limmathurotsakul, Direk; Mahidol Oxford Tropical Medicine Research Unit; University of Oxford, Centre for Tropical Medicine and Global Health, Nuffield Department of Medicine<br>Karkey, Abhilasha; University of Oxford Nuffield Department of Clinical Medicine, Centre for Tropical Medicine and Global Health; Oxford University Clinical Research Unit, Nepal Unit<br>Dodds Ashley, Elizabeth; Duke University, Duke Antimicrobial Stewardship Outreach Network, Duke Center for Antimicrobial Stewardship and Infection Prevention<br>Anderson, D; Duke University, Duke Antimicrobial Stewardship Outreach Network, Duke Center for Antimicrobial Stewardship and Infection Prevention<br>Patel, Payal ; Centers for Disease Control and Prevention<br>Patel, Twisha ; Centers for Disease Control and Prevention<br>Lessa, Fernanda; Centers for Disease Control and Prevention<br>van Doorn, H. Rogier; Oxford University Clinical Research Unit; University of Oxford Nuffield Department of Clinical Medicine
**Primary Subject Heading**:	Health services research
Secondary Subject Heading:	Health policy, Public health
Keywords:	HEALTH SERVICES ADMINISTRATION & MANAGEMENT, INFECTIOUS DISEASES, PUBLIC HEALTH, Quality in health care < HEALTH SERVICES ADMINISTRATION & MANAGEMENT

SCHOLARONE™
Manuscripts

Manuscript Title: Identifying context-specific domains for assessing antimicrobial stewardship programs in Asia: Protocol for a scoping review

Authors: Vu Thi Lan Huong[1]*, Raph L. Hamers[2,3], Ralalicia Limato[2,3], Direk Limmathurotsakul[3,4], Abhilasha Karkey[3,5], Elizabeth Dodds-Ashley[6], Deverick Anderson[6], Payal K. Patel[7], Twisha S. Patel[7], Fernanda C. Lessa[7], H Rogier van Doorn[1,3]

Affiliations:

[1]Oxford University Clinical Research Unit – Ha Noi, Viet Nam, 78 Giai Phong – Dong Da – Ha Noi – Viet Nam

[2]Eijkman-Oxford Clinical Research Unit – Jakarta, Indonesia

[3]Centre for Tropical Medicine and Global Health, Nuffield Department of Medicine, University of Oxford, Oxford, UK

[4]Mahidol Oxford Tropical Medicine Research Unit – Bangkok, Thailand

[5]Oxford University Clinical Research Unit - Kathmandu, Nepal

[6]Duke Antimicrobial Stewardship Outreach Network, Duke Center for Antimicrobial Stewardship and Infection Prevention, Duke University, Durham, NC 27710, United States

[7]Division of Healthcare Quality Promotion, US Centers for Disease Control and Prevention, USA

Correspondence to Dr Vu Thi Lan Huong: huongvtl@oucru.org

CDC disclaimer: The findings and conclusions in this report are those of the author(s) and do not necessarily represent the official position of the U.S. Centers for Disease Control and Prevention

For peer review only

Abstract

Introduction

Antimicrobial stewardship (AMS) is an important strategy to control antimicrobial resistance. Resources are available to provide guidance for design and implementation of AMS programs, however these may have limited applicability in resource-limited settings including those in Asia. This scoping review aims to identify context-specific domains and items for the development of a healthcare-facility (HCF)-level tool to guide AMS implementation in Asia.

Methods and analysis

This review is the first step in a larger project to assess AMS implementation, needs and gaps in Asia. We will employ a deductive qualitative approach to identify locally appropriate domains and items of AMS implementation guided by Nilsen & Bernhardsson's contextual dimensions. This process is also informed by discussions from a technical advisory group coordinated by the U.S. Centers for Disease Control and Prevention to develop an AMS HCF-level assessment tool for low- and middle-income countries (LMIC). We will review English-language documents that discuss HCF-level implementation, including those describing frameworks, components/elements, or recommendations for design, implementation or assessment globally and specific to Asia. We have performed the search in August-September 2021 including general electronic databases (MEDLINE, Embase, Web of Science and Google Scholar), region-specific databases, national action plans, grey literature sources and reference lists to identify eligible documents. Country-specific documents will be restricted to countries in three sub-regions: South Asia, East Asia and Southeast Asia. Codes and themes will be derived through a content analysis, classified following the predefined context dimensions and used for developing domains and items of the assessment tool.

Ethics and dissemination

Results from this review will feed into our stepwise process for developing a context-specific HCF-level assessment tool for AMS programs to assess the implementation status, identify intervention opportunities and monitor progress over time. The process will be done in consultation with local stakeholders, the end-users of the generated knowledge.

For peer review only

Strengths and limitations of this review

- This scoping review will be guided by determinant frameworks that identify the context dimensions of implementation and by experts and current research to provide comprehensive and in-depth description of the relevant aspects of AMS programs in general and in Asia;
- We will employ a combination of search strategies that includes different types of databases to efficiently identify a broad spectrum of guidance documents from published and grey literature in English;
- Local experts including academics and practitioners in AMS in Asia who are the end-users of this generated knowledge will be engaged during the review process;
- We will not include original studies that reported results of AMS implementation from a limited number of single institutions or locations (due to the vast number of such reports)

For peer review only

Introduction

The prevalence and incidence rates of antimicrobial resistant (AMR) infections continue to rise, with higher levels of resistance reported from low-and middle-income countries (LMIC) compared to high-income countries. [1] Misuse and overuse of antimicrobials in healthcare continues to be one major driver as consumption is accelerating worldwide especially in emerging economies. At the same time, access to life-saving drugs targeting the emerging resistant pathogens remains an issue in these settings while development of new drugs has been slow especially for the antibiotics against gram-negative pathogens. [2]

In this context, antimicrobial stewardship (AMS) is an important strategy in global and national action plans to control AMR. This has been defined in a number of ways from technical descriptions focusing on prescriptions (e.g., drug, dose, duration) to the concepts of responsibility in how antibiotics are used (e.g., careful and responsible decisions to treat). [3] AMS has also been expanded beyond its core strategies and adult inpatient settings, with increasing roles of front-line providers (e.g., nurses and pharmacists), rapidly evolving metrics for impact and success, and wide spectrum of implementation, engagement and practice. [4]

Guidance for AMS programs are available from many resources such as those from World Health Organization (WHO) and the U.S. Centers for Disease Control and Prevention (CDC) [5-7], expert reviews [8 9], country and region specific guidelines. The core elements and components of AMS programs identified by several groups [10-13] often share similar aspects. These include senior management commitment, resource allocation, leadership, accountability, expertise on infection management and antimicrobials, specific actions to improve antimicrobial use, education and training, monitoring use and resistance epidemiology, and reporting/feedback. These existing guidance and tools are usually not context specific, not tailored for resource-limited settings, and not focused on implementation aspects of the programs. The WHO toolkit for LMICs issued in 2019 and WHO policy guidance on integrated antimicrobial stewardship activities issued in May 2021 discuss different sets of measures/indicators for assessing program impact at the healthcare facility (HCF) level [7 14], which include structural, process and outcome measures but these assessments are at a high level and do not include specific implementation contextual details of stewardship interventions in these settings. Therefore, existing

For peer review only

guidance resources should be reviewed to develop a healthcare-facility (HCF)-level tool that will guide implementation of AMS in hospitals in Asia.

Review rationale

The existing guidance documents may have limited applicability to many countries in Asia where healthcare systems, the availability of diagnostic testing and antibiotics, public awareness and prescribing practices [8 15], and contextual and cultural characteristics [16 17] differ from the other regions. Therefore, contextualization of the assessment of the status of AMS program implementation is important. A “glocalization” approach has been discussed for reconciliation of global frameworks and methodologies and the local contexts in AMS implementation [18]. In November 2016, an expert panel consisting of 11 infectious diseases experts, researchers and opinion leaders from Asia developed a consensus statement on AMS programs for acute care hospitals in the Asian region [19]. The panel identified common gaps and challenges facing AMS programs, potential solutions, and process and outcome measures for evaluating impact of the programs. A checklist was designed based on existing materials. However, it provides limited in-depth understanding of the current state to support effective planning of targeted interventions. In May 2021, WHO issued a policy guidance on integrated AMS activities [14]. Within this guidance, WHO developed a list of essential national and HCF core elements to assist with strengthening AMS structures at both levels. Context-specific matters for HCF-level AMS implementation are not yet captured in the HCF tool. Understanding implementation needs and practices within the Asian context and being able to monitor progress over time are key to assist local healthcare facilities with improving antimicrobial use. A context-specific assessment tool that is practical and responsive to the local conditions is needed to support institutions to start and maintain AMS programs in Asia. This assessment tool will be complementary to the WHO tools on national and HCF-level to provide support for hospitals in assessing their AMS status and improving the program implementation.

Review objectives

This scoping review protocol aims to describe our methods to identify the context-specific domains and items to develop a tailored assessment tool for Asia. Existing guidance documents will be analyzed to extract these contextual domains and items related to an AMS program in general and specific to Asian settings. This work constitutes the first step in a stepwise approach to the development of an integrated

For peer review only - http://bmjopen.bmj.com/site/about/guidelines.xhtml

assessment tool that can be used to assess the current practices and needs and identify priorities for implementation and improvement of AMS programs in Asia.

Methods and analysis

Conceptual model

To guide our data extraction and understanding of contextual features in AMS implementation, we will use the four levels of contextual dimensions (micro, meso, macro, and multiple) as identified by Nilsen & Bernhardsson in their review of contextual determinants for implementation outcomes from 17 determinant frameworks [20]. We will employ a deductive qualitative approach to identify the domains and items of AMS implementation according to the four contextual dimensions as a framework for reference (Table 1). This process is also informed by the discussions from a technical advisory group coordinated by the U.S. CDC that has been formed to develop a HCF assessment tool focused on AMS implementation.

Table 1. List of contextual dimensions for program implementation to guide the scoping review of AMS guidance documents

Dimension	Description of initial themes and codes for data extraction
Micro level of healthcare	Patients' factors (knowledge, attitudes, preferences, needs, and resources in antibiotic use and AMR)
Meso level of healthcare	Organizational culture and climate, readiness to change (commitment, preparation, prioritization, efficacy and capacity to change, tension, practicality, flexibility), support (administration, staff, training resources, information and decision-support systems, expert support), structures (size, complexity, specialization, differentiation, decentralization)
Macro level of healthcare	Exogenous influences (policies, guidelines, research evidence, regulation and legislation, mandates, directives, recommendations, political stability, public reporting, benchmarking, organizational networks)
Multiple levels of healthcare	Social relations and support, financial resources (funding, reimbursement, incentives, costs), leadership (leaders, champions…), time restrictions, feedback (mechanisms for

For peer review only - http://bmjopen.bmj.com/site/about/guidelines.xhtml

	monitoring and providing feedback), physical conditions (equipment, facilities)

Protocol design

This protocol follows the methodology for scoping review developed by Arksey and O'Malley [21] and Levac *et al* [22] through six steps:

Stage 1: Identifying the research question

This scoping review is designed to answer the following question: “What are the domains and items that can constitute an assessment tool of AMS implementation in Asia at a HCF level?” The assessment tool will aim to assess the state of AMS implementation in healthcare facilities and contextual factors can influence its success and impact. It will also aim to identify challenges and opportunities, and help formulate priorities by generating actionable recommendations for next steps in the implementation of AMS programs. Based on the conceptual model (Table 1), we will identify the specific domains and items of four contextual dimensions that are important and relevant for AMS assessment and improvement at the HCF in Asia. These domains and items will also be sent to a group of local experts in Asia for consultation on their relevance.

Stage 2: Identifying relevant documents

We will search a number of electronic databases of published and unpublished literature to identify documents that provide guidance on AMS implementation globally and specific to Asia (Table 2). Country-specific documents will be restricted to the countries within the three sub-regions of Asia: South Asia, East Asia and Southeast Asia. After this search, the study investigator group will review the search results to determine if documents identified have included the available AMS guidance resources commonly used and those that are mentioned to a lesser extent in the literature to make sure the search strategy has been appropriate and comprehensive. The search will be expanded to additional search engines available and manually checking all reference lists of included documents will also be performed to identify additional documents of relevance. We will also conduct a targeted search of the grey literature in local and international organisations' websites.

Table 2. List of databases to be searched for the scoping review

For peer review only

Database category	Databases
General database (a recommended optimal combination for searching systematic reviews [23])	Embase<br>MEDLINE (including “Epub Ahead of Print, In-Process, and Other Non-Indexed Citations”)<br>Web of Science (Core Collection)<br>Google Scholar (first 200 references of relevance)
Regional database	China: Chinese biomedical literature Database (CBM)<br>India: IndMED<br>Korea: KoreaMed<br>South-east Asia: Index Medicus for the South-East Asia Region (IMSEAR)<br>China National Knowledge Infrastructure (CNKI)<br>Chinese Scientific Journal Database (VIP)<br>SaudiMedLit<br>Thai Index Medicus<br>Thai Journal Citation Index Centre<br>Thai Medical Index<br>Western Pacific Region Index Medicus (WPRIM)<br>WANFANG Data<br>Korea: RISS- Korean Education and Research Information Service<br>Asia: Bibliography of Asian Studies<br>Health Research and Development Information Network (HERDIN)<br>Japan: Cinii<br>Indonesia: Garba Rujukan Digital, Neliti
Grey literature database	ProQuest Dissertation and Theses Database (PQDT)<br>OpenGrey<br>Grey literature report

Following search terms will be used for the general databases (Embase MEDLINE, Web of Science, Google Scholar) as both keywords in the title and/or abstract and subject headings (eg, MeSH, EMTREE) as appropriate: (“antimicrobial stewardship” OR “antibiotic stewardship”) AND (“framework” OR “guid*” OR “tool*” OR “recommend*” OR “step” OR “approach” OR “policy”). For other databases, we

For peer review only - http://bmjopen.bmj.com/site/about/guidelines.xhtml

will search using less restrictive terms ("antimicrobial stewardship" OR "antibiotic stewardship") to be able to identify local relevant documents. Only documents published in English language will be included, with no restriction on years of publication.

Stage 3: Selection of documents for review

This stage will be an iterative process: searching, refining the search strategy, and reviewing articles for eligibility and inclusion into the final list of documents. The team will meet to discuss inclusion and exclusion criteria at the beginning of the process, and to review challenges and uncertainties and to refine the search strategy as needed along the process. The review process will essentially involve two steps. In the first step, two research assistants will independently screen the titles and abstracts of all citations returned from the search databases. Any documents considered relevant by either or both researchers will be included in the list for full-text screening at this step. In step two, the lead investigator (VTLH) together with the two research assistants will review the full-text of these documents and select those that fully meet inclusion/exclusion criteria for data extraction stage. Any discrepancies the documents selected arise during the process will be discussed, and decision will be made through consensus within the team.

Documents will be included if they provide guidance for and/or input information that can help inform AMS implementation at the HCF level, including those describing frameworks, program components and elements, or recommendations for design, implementation or assessment. We will exclude any documents that only describe methods or results of research studies or reports on AMS programs implemented at single healthcare facilities with a limited geographical scope. We will also exclude documents without identifiable authors, publishers, or year of publication. For guidance documents from the same institutions that were updated over time, we will only include the most updated version in the review.

Stage 4: Data extraction

Characteristics of included documents will be extracted using a data extraction form, which will be reviewed and updated along the extraction process as appropriate. Following information will be captured: confirmation on document's inclusion/ exclusion criteria, publication year, geographical scope (global or regional), country, type of document (guidance, review, perspective/commentary, others), author,

For peer review only

publisher and type (government agency, international body, expert group, individual authors, not-for-profit organization, for-profit organization, others). Data extraction will be based on the content published, no attempts will be made to contact authors for clarifications.

The content of the included documents will be read in-depth and coded following the context dimensions described by Nilsen & Bernhardsson. Initial themes and codes were developed based on the contextual dimensions as described in Table 1. Codes are the specific summarizing phrases; each code presents one specific area of AMS implementation that should be assessed. Themes are the higher-order categories consisting of multiple codes, each theme represents one broad area or construct of AMS implementation. The initial structure of themes and codes can be expanded during the extraction to add additional codes and themes and can be re-structured if needed for clarity. We will use a deductive qualitative content analysis [24] approach that allows the analysis to evolve from our existing understanding of AMS implementation and the existing research about program implementation. We will use NVivo software, a package for text-based analysis, to manage the texts and systematically organize our reading and coding. The qualitative content analysis is a popular, flexible, and pragmatic method of analyzing text data to attain a condensed and broad description of the topic through a systematic classification process involving coding and identifying themes and patterns [24]. The coding process will be conducted with close supervision by two experienced researchers of the research team. The research team will meet regularly to review the codes identified and ensure data extraction is consistent with the research question and purpose.

Stage 5: Data summary and synthesis of results

After the documents were read and coded, we will summarize and organize the codes and themes into a cohesive and coherent structure. From this, we will identify possible items and domains that will be used to develop a HCF-level AMS assessment tool of AMS implementation. One item can be identified from the content of multiple codes and presented in a question format. One domain can be identified from the content of multiple themes that can help describe the items under it. Through this scoping review process, we will be able to achieve a broad and in-depth description of the various aspects of AMS implementation that are applicable to Asian settings. This will help identify aspects commonly encountered in an AMS program, but also point out the region-specific issues, challenges and opportunities

For peer review only - http://bmjopen.bmj.com/site/about/guidelines.xhtml

and help the development of a practical and responsive assessment tool for use in these settings.

Stage 6: Consultation

As this review aims to identify appropriate domains and items for the assessment tool specific to Asia, consultation with local experts on the identified content is an important step to check for validity and relevance. We will engage local experts who have experience with AMS in the region including academics and practitioners who will be the end-users of the knowledge generated from this scoping review. These local experts will be identified through our own individual networks in four countries where the investigator group are working in (Indonesia, Nepal, Thailand and Vietnam) and from the published literature during our search. The identification and selection of the local experts is purposive in order to engage those with the most appropriate experience. The feedback from these local experts on the list of domains and items will be captured using a web-based survey. With the expected return rate of 30%, we plan to approach up to 90 potential experts (60 from the local experts in four countries and 30 from the literature.)

Patient and Public Involvement

Patients or the public will not be involved in this scoping review. The assessment domains and items identified from this scoping review will be sent to local experts in antimicrobial stewardship for their feedback on the relevance of these to be included in the assessment tool at healthcare facility level.

Dissemination and ethics

This scoping review is the first step in the overall project coordinated by the U.S. CDC to assess AMS implementation in LMICs including Asia. The results from this scoping review will be used to develop a context-specific, assessment tool aiming at characterizing the current state of AMS implementation and identifying interventions and opportunities for improvement. The scoping review process will only involve identification, selection and analysis of documents available for use in the public domains, therefore an ethics approval is not required in this study. The whole process of assessment tool development will be done in consultation with local stakeholders who are the end-users of the generated knowledge.

For peer review only - http://bmjopen.bmj.com/site/about/guidelines.xhtml

Contributors: VTLH conceived of the idea, developed the research question and study methods. RLH, RL, DL, AK, EDA, DA, PKP, TSP, FCL and HRvD reviewed and aided in developing the research question and study methods. HRvD supervised the work throughout. All authors contributed meaningfully to the drafting and editing, and approved the final manuscript.

Funding: This work is part of the project entitled "Understanding variations in antimicrobial stewardship (AMS) programs in hospital networks in Asia through a newly developed context-specific tool" funded by U.S. Centers for Disease Control and Prevention (CDC) through the Broad Agency Announcement (BAA) 75D301-21-R-71738. HVTL was also supported by the National Institute for Health Research (NIHR) (using the UK's Official Development Assistance (ODA) Funding) and Wellcome (Grant Reference Number: 216367/Z/19/Z) under the NIHR-Wellcome Partnership for Global Health Research. The views expressed are those of the authors and not necessarily those of Wellcome, the NIHR or the Department of Health and Social Care.

Competing interests: None declared.

Data sharing statement: No additional data are available.

ORCID IDs:
Vu Thi Lan Huong 0000-0002-9579-5576
H. Rogier van Doorn 0000-0002-9807-1821
Raph L. Hamers 0000-0002-5007-7896
Direk Limmathurotsakul 0000-0001-7240-5320
Ralalicia Limato 0000-0002-5306-3254

For peer review only

References

1. Temkin E, Fallach N, Almagor J, et al. Estimating the number of infections caused by antibiotic-resistant *Escherichia coli* and *Klebsiella pneumoniae* in 2014: a modelling study. *Lancet Glob Health* 2018;6(9):e969-e79.

2. Sriram A, Kalanxhi E, Kapoor G, et al. State of the world's antibiotics 2021: A global analysis of antimicrobial resistance and its drivers. Washington DC.: Center for Disease Dynamics, Economics & Policy, 2021.

3. Dyar OJ, Huttner B, Schouten J, et al. What is antimicrobial stewardship? *Clin Microbiol Infect* 2017;23(11):793-98.

4. Emberger J, Tassone D, Stevens MP, et al. The Current State of Antimicrobial Stewardship: Challenges, Successes, and Future Directions. *Current infectious disease reports* 2018;20(9):31.

5. Centers for Disease Control and Prevention (CDC). Core Elements of Hospital Antibiotic Stewardship Programs, https://www.cdc.gov/antibiotic-use/core-elements/hospital.html. 2019

6. Centers for Disease Control and Prevention. Core elements of antibiotic stewardship programs in resource-limited settings. Atlanta, GA, 2018.

7. World Health Organization. Antimicrobial stewardship programmes in health-care facilities in low- and middle-income countries: a WHO practical toolkit. Geneva: World Health Organization, 2019.

8. Resman F. Antimicrobial stewardship programs; a two-part narrative review of step-wise design and issues of controversy Part I: step-wise design of an antimicrobial stewardship program. *Ther Adv Infect Dis* 2020;7:2049936120945083.

9. Mendelson M, Morris AM, Thursky K, et al. How to start an antimicrobial stewardship programme in a hospital. *Clin Microbiol Infect* 2020;26(4):447-53.

10. Pulcini C, Binda F, Lamkang AS, et al. Developing core elements and checklist items for global hospital antimicrobial stewardship programmes: a consensus approach. *Clin Microbiol Infect* 2019;20(1):20-25. doi: 10.1016/j.cmi.2018.03.033

11. Pollack LA, Srinivasan A. Core elements of hospital antibiotic stewardship programs from the Centers for Disease Control and Prevention. *Clin Infect Dis* 2014;59 Suppl 3(Suppl 3):S97-100.

12. Pollack LA, Plachouras D, Gruhler H, et al. Summary the modified Delphi process for common structure and process indicators for hospital antimicrobial stewardship programs (Transatlantic Taskforce on Antimicrobiol Resistance). 2015. https://www.cdc.gov/drugresistance/pdf/summary_of_tatfar_recommendation_1.pdf (accessed 16 January 2022).

For peer review only - http://bmjopen.bmj.com/site/about/guidelines.xhtml

13. European Commission. EU Guidelines for the prudent use of antimicrobials in human health. *Official Journal of the European Union* 2017;2017/C 212/01

14. World Health Organization. WHO policy guidance on integrated antimicrobial stewardship activities. Geneva: World Health Organization, 2021.

15. Cox JA, Vlieghe E, Mendelson M, et al. Antibiotic stewardship in low- and middle-income countries: the same but different? *Clin Microbiol Infect* 2017;23(11):812-18.

16. Charani E, Smith I, Skodvin B, et al. Investigating the cultural and contextual determinants of antimicrobial stewardship programmes across low-, middle- and high-income countries-A qualitative study. *PLoS One* 2019;14(1):e0209847.

17. Gebretekle G, Haile Mariam D, Abebe W, et al. Opportunities and barriers to implementing antibiotic stewardship in low and middle-income countries: Lessons from a mixed-methods study in a tertiary care hospital in Ethiopia. *PLoS One* 2018;13(12):e0208447.

18. Rubin O. The glocalization of antimicrobial stewardship. *Global Health* 2019;15(1):54-54.

19. Apisarnthanarak A, Kwa AL-H, Chiu C-H, et al. Antimicrobial stewardship for acute-care hospitals: An Asian perspective. *Infect Control Hosp Epidemiol* 2018;39(10):1237-45.

20. Nilsen P, Bernhardsson S. Context matters in implementation science: a scoping review of determinant frameworks that describe contextual determinants for implementation outcomes. *BMC Health Services Research* 2019;19(1):189.

21. Arksey H, O'Malley L. Scoping studies: towards a methodological framework. *International Journal of Social Research Methodology* 2005;8(1):19-32.

22. Levac D, Colquhoun H, O'Brien KK. Scoping studies: advancing the methodology. *Implementation Science* 2010;5(1):69.

23. Bramer WM, Rethlefsen ML, Kleijnen J, et al. Optimal database combinations for literature searches in systematic reviews: a prospective exploratory study. *Systematic Reviews* 2017;6(1):245.

24. Hsieh H-F, Shannon SE. Three Approaches to Qualitative Content Analysis. *Qualitative Health Research* 2005;15(9):1277-88.

For peer review only - http://bmjopen.bmj.com/site/about/guidelines.xhtml

Preferred Reporting Items for Systematic reviews and Meta-Analyses extension for Scoping Reviews (PRISMA-ScR) Checklist

SECTION	ITEM	PRISMA-ScR CHECKLIST ITEM	REPORTED ON PAGE #
**TITLE**			
Title	1	Identify the report as a scoping review.	1
**ABSTRACT**			
Structured summary	2	Provide a structured summary that includes (as applicable): background, objectives, eligibility criteria, sources of evidence, charting methods, results, and conclusions that relate to the review questions and objectives.	2
**INTRODUCTION**			
Rationale	3	Describe the rationale for the review in the context of what is already known. Explain why the review questions/objectives lend themselves to a scoping review approach.	5
Objectives	4	Provide an explicit statement of the questions and objectives being addressed with reference to their key elements (e.g., population or participants, concepts, and context) or other relevant key elements used to conceptualize the review questions and/or objectives.	5-7
**METHODS**			
Protocol and registration	5	Indicate whether a review protocol exists; state if and where it can be accessed (e.g., a Web address); and if available, provide registration information, including the registration number.	NA
Eligibility criteria	6	Specify characteristics of the sources of evidence used as eligibility criteria (e.g., years considered, language, and publication status), and provide a rationale.	7-9
Information sources*	7	Describe all information sources in the search (e.g., databases with dates of coverage and contact with authors to identify additional sources), as well as the date the most recent search was executed.	7-9
Search	8	Present the full electronic search strategy for at least 1 database, including any limits used, such that it could be repeated.	7-8
Selection of sources of evidence†	9	State the process for selecting sources of evidence (i.e., screening and eligibility) included in the scoping review.	9
Data charting process‡	10	Describe the methods of charting data from the included sources of evidence (e.g., calibrated forms or forms that have been tested by the team before their use, and whether data charting was done independently or in duplicate) and any processes for obtaining and confirming data from investigators.	9-10
Data items	11	List and define all variables for which data were sought and any assumptions and simplifications made.	9
Critical appraisal of individual sources of evidence§	12	If done, provide a rationale for conducting a critical appraisal of included sources of evidence; describe the methods used and how this information was used in any data synthesis (if appropriate).	NA

For peer review only - http://bmjopen.bmj.com/site/about/guidelines.xhtml

SECTION	ITEM	PRISMA-ScR CHECKLIST ITEM	REPORTED ON PAGE #
Synthesis of results	13	Describe the methods of handling and summarizing the data that were charted.	10
**RESULTS**			
Selection of sources of evidence	14	Give numbers of sources of evidence screened, assessed for eligibility, and included in the review, with reasons for exclusions at each stage, ideally using a flow diagram.	NA
Characteristics of sources of evidence	15	For each source of evidence, present characteristics for which data were charted and provide the citations.	NA
Critical appraisal within sources of evidence	16	If done, present data on critical appraisal of included sources of evidence (see item 12).	NA
Results of individual sources of evidence	17	For each included source of evidence, present the relevant data that were charted that relate to the review questions and objectives.	NA
Synthesis of results	18	Summarize and/or present the charting results as they relate to the review questions and objectives.	NA
**DISCUSSION**			
Summary of evidence	19	Summarize the main results (including an overview of concepts, themes, and types of evidence available), link to the review questions and objectives, and consider the relevance to key groups.	NA
Limitations	20	Discuss the limitations of the scoping review process.	NA
Conclusions	21	Provide a general interpretation of the results with respect to the review questions and objectives, as well as potential implications and/or next steps.	NA
**FUNDING**			
Funding	22	Describe sources of funding for the included sources of evidence, as well as sources of funding for the scoping review. Describe the role of the funders of the scoping review.	11

JBI = Joanna Briggs Institute; PRISMA-ScR = Preferred Reporting Items for Systematic reviews and Meta-Analyses extension for Scoping Reviews.

* Where *sources of evidence* (see second footnote) are compiled from, such as bibliographic databases, social media platforms, and Web sites.

† A more inclusive/heterogeneous term used to account for the different types of evidence or data sources (e.g., quantitative and/or qualitative research, expert opinion, and policy documents) that may be eligible in a scoping review as opposed to only studies. This is not to be confused with *information sources* (see first footnote).

‡ The frameworks by Arksey and O'Malley (6) and Levac and colleagues (7) and the JBI guidance (4, 5) refer to the process of data extraction in a scoping review as data charting.

§ The process of systematically examining research evidence to assess its validity, results, and relevance before using it to inform a decision. This term is used for items 12 and 19 instead of "risk of bias" (which is more applicable to systematic reviews of interventions) to include and acknowledge the various sources of evidence that may be used in a scoping review (e.g., quantitative and/or qualitative research, expert opinion, and policy document).

From: Tricco AC, Lillie E, Zarin W, O'Brien KK, Colquhoun H, Levac D, et al. PRISMA Extension for Scoping Reviews (PRISMAScR): Checklist and Explanation. Ann Intern Med. 2018;169:467–473. doi: 10.7326/M18-0850.

For peer review only - http://bmjopen.bmj.com/site/about/guidelines.xhtml
